# Circulating Tumor DNA Analyses Predict Disease Recurrence in Non-Muscle-Invasive Bladder Cancer

**DOI:** 10.3389/fonc.2021.657483

**Published:** 2021-04-28

**Authors:** Jinghua Zhang, Daofeng Dai, Junqiang Tian, Lifeng Li, Jing Bai, Yaping Xu, Zhiping Wang, Aifa Tang

**Affiliations:** ^1^ College of Life Sciences, Henan Agricultural University, Zhengzhou, China; ^2^ Health Science Center, The First Affiliated Hospital of Shenzhen University, and Guangdong Key Laboratory of Systems Biology and Synthetic Biology for Urogenital Tumors, Shenzhen Second People’s Hospital, Shenzhen, China; ^3^ Jiangxi Otorhinolaryngology Head and Neck Surgery Institute, Department of Otorhinolaryngology Head and Neck Surgery, The First Affiliated Hospital of Nanchang University, Nanchang, China; ^4^ Key Laboratory of Gansu Province for Urological Diseases, Department of Urology of Lanzhou University Second Hospital, Urology Institute of Lanzhou University Second Hospital, Lanzhou, China; ^5^ Geneplus-Beijing Institute, Beijing, China; ^6^ Shenzhen Luohu Hospital Group, The Third Affiliated Hospital of Shenzhen University, Shenzhen, China

**Keywords:** non-muscle-invasive bladder cancer, circulating tumor DNA, targeted sequencing, prognostic biomarker, chemotherapy, molecular tumor burden index

## Abstract

Circulating tumor DNA (ctDNA) can be a prognostic biomarker for non-muscle-invasive bladder cancer (NMIBC); however, targeted sequencing has not been performed to detect ctDNA in NMIBC. We applied targeted sequencing based on an 861-gene panel to determine mutations in tumor tissue DNA and plasma ctDNA in 82 NMIBC patients receiving transurethral resection (TUR) of bladder followed by immunotherapy. We detected 476 and 165 somatic variants in tumor DNA from 82 NMIBC patients (100%) and ctDNA from 54 patients (65.85%), respectively. Patients with high heterogeneity in tumor DNA had a significantly shorter disease-free survival than those with low heterogeneity. Tumor-derived alterations were detectable in plasma of 43 patients (52.44%). The concordance of somatic variants between tumor DNA and plasma ctDNA were higher in patients with T1 stage (p < 0.0001) and tumor size ≥3 cm (p = 0.0002). Molecular tumor burden index (mTBI) in ctDNA positively correlated with larger tumor size (p = 0.0020). A higher mTBI was an independent predictor of recurrence after TUR of bladder followed by immunotherapy. Analysis of ctDNA based on targeted sequencing is a promising approach to predict disease recurrence for NMIBC patients receiving TUR of bladder followed by immunotherapy.

## Introduction

Bladder cancer is a common malignant tumor, with an estimated 549,000 new cases and 200,000 deaths worldwide every year (1). The most common histological type of bladder cancer is urothelial carcinoma (91.4%), which can be divided into non-muscle-invasive bladder cancer (NMIBC) and muscle-invasive bladder cancer (MIBC), accounting for 74 and 25.2%, respectively (2). NMIBC is characterized by a high recurrence rate of 50–70% and a 5-year survival rate of 90% (3). Progression to MIBC is observed in 20–30% of patients with NMIBC (4). Ultimately, as many as 10–15% of NMIBC patients will die of bladder cancer (5). Thus, it is urgent to develop a biomarker for discriminating high-risk from low-risk NMIBC patients, and for prediction of disease recurrence.

In the past 5 years, considerable advancements have been made with liquid biopsy, which is an important method in precision medicine, especially in oncology. The advantage of liquid biopsy is that it only requires the patient’s blood for non-invasive detection without invasive harvesting of the patient’s tissue and can monitor gene mutations in patients in real time (6, 7). Circulating tumor DNA (ctDNA) is a DNA fragment secreted into peripheral blood by apoptotic tumor cells (8). ctDNA only constitutes a small fraction of circulating cell-free DNA (cfDNA) in peripheral blood; the majority of cfDNA stems from leukocytes. Therefore, it is difficult to detect somatic variants in ctDNA, especially for early-stage cancers.

Currently, plasma ctDNA has exhibited great potential as a biomarker in several cancers, particularly in advanced metastatic cancer. For example, a high concordance rate of the mutation status and changes in copy number between liquid and solid biopsy have been reported for metastatic castration-resistant prostate cancer (9). The European Medicines Agency approved liquid biopsy for epidermal growth factor receptor for lung cancer patients (10, 11). However, there are only two reports on ctDNA in NMIBC. Birkenkamp-Demtröder et al. established methods for non-invasive disease surveillance in patients with NMIBC, using highly sensitive and tumor-specific personalized plasma- and urine-based assays, and demonstrated that somatic variants of ctDNA were detectable in plasma and urine samples from patients with NMIBC (12). Christensen et al. developed droplet digital polymerase chain reaction (ddPCR) analyses and screened ctDNA for FGFR3 and PIK3CA hotspot mutations in urine and plasma from NMIBC and MIBC patients undergoing radical cystectomy (13). They demonstrate that increased levels of FGFR3 and PIK3CA mutated DNA in urine and plasma are indicative of later progression and metastasis in bladder cancer. Nevertheless, these two studies both utilized ddPCR assays, and whether next-generation sequencing (NGS) approaches can be applied in screening ctDNA for somatic variations in plasma from NMIBC patients has not been explored.

In this study, we performed targeted sequencing based on an 861-gene panel to screen matched plasma ctDNA and tumor DNA for somatic mutations in patients with NMIBC, evaluated the effects of clinicopathological features on the concordance of somatic variants between ctDNA and tumor DNA, and analyzed whether ctDNA could be a biomarker for prediction of disease recurrence after transurethral resection (TUR) of bladder followed by immunotherapy.

## Materials and Methods

### Patients and Sample Collection

This study included 82 NMIBC patients who underwent TUR of bladder followed by bladder transfusion immunotherapy [agent: Bacille Calmette-Guérin (BCG)]; mode of administration: bladder transfusion immunotherapy; number of doses/cycles: the period of perfusion is usually once a week, a total of six to eight times, and then once a month, a total of 10 times) to avoid recurrence between May 2015 and June 2016 at Lanzhou University Second Hospital. Archival slides of patients were evaluated by two pathologists. We obtained the following clinicopathological information: gender, age upon diagnosis, tumor grade, tumor size, and tumor stage. The clinicopathological information was supplied in **Table 1**. Approval for this study was obtained from Medical Ethics Committee of Shenzhen Second People’s Hospital. Patients signed an informed consent. The study was conducted in compliance with the principles of the Declaration of Helsinki and local ethical and legal requirements.

**Table 1:** Summary of clinical features and sequencing parameters in patients with non-muscle-invasive bladder cancer.

Parameter	Number (%)
Median age (range), y	63.5 (32–83)
Gender	
Female	13 (15.85%)
Male	69 (84.15%)
T stage	
Ta	48 (58.54%)
TI	34 (41.46%)
Grade	
Low	42 (51.22%)
High	40 (48.78%)
Tumor size	
<3 cm	57 (69.51%)
≥3 cm	25 (30.49%)
Median cfDNA yield (range), ng/ml	31.2 (5.49–458.67)
Median sequencing depth (range), ×	
Tumor DNA	940× (421–1,656×)
cfDNA^†^	1,199× (404–3,965×)

^†^cfDNA circulating, cell-free DNA.

### DNA Extraction

Tumor tissue samples were obtained by TUR of bladder, and at least 10 ml of Peripheral blood was collected in an EDTA tube prior to surgery, centrifuged twice at 4°C for 10 min (3,000 and 15,000 revolutions per minute). Tissues, plasma, and peripheral blood lymphocytes (PBLs) were stored at −80°C prior to DNA extraction. cfDNA was extracted from plasma samples with the QIAamp Circulating Nucleic Acid Kit (Qiagen, Hilden, Germany). DNA of PBLs was extracted from peripheral blood cells using the QIAamp DNA Blood Mini Kit (Qiagen). Tissue DNA was extracted using the QIAamp DNA Mini Kit (Qiagen). All DNA was extracted according to the manufacturer’s instructions. DNA of PBLs was sequenced as the normal control sample.

### Targeted Capture, and Next-Generation Sequencing

DNA samples were used to construct libraries and perform targeted capture sequencing. To design a capture region for bladder cancer, we selected 861 genes covering recurrent mutations from the Catalogue of Somatic Mutations in Cancer; known oncogenes and tumor suppressor genes; genes in key signaling pathways related to the bladder cancer.

Sequencing libraries were constructed with the KAPA DNA Library Preparation Kit (Kapa Biosystems, Wilmington, MA, USA) following the manufacturer’s protocol. Libraries were hybridized to custom-designed biotinylated oligonucleotide probes (Integrated DNA Technologies, Iowa, IA, USA). DNA sequencing was carried out using the HiSeq 3000 Sequencing System (Illumina, San Diego, CA, USA) with 2×101-bp paired-end reads.

### Sequencing Data Analysis

Terminal adaptor sequences and low-quality reads were removed from raw data. BWA (version 0.7.12-r1039) was employed to align the clean reads to the reference human genome (hg19). Picard (version 1.98) was used to mark PCR duplicates. Realignment and recalibration were performed using GATK (version 3.4-46-gbc02625). Single nucleotide variants (SNVs) were called using MuTect2 (version 3.5.0). Small insertions and deletions (Indels) were also called using MuTect2. Variants detected in matched control DNA samples with one or more reads indicative of indels at the same location or in the 40-bp flanking regions of experimental samples or residing near regions with low complexity or short tandem repeats were filtered. Somatic copy-number alterations were identified with CONTRA (version 2.0.8) (14). The final candidate variants were all manually verified in the Integrative Genomics Viewer (IGV) (15).

### Statistical Analysis

All statistical analyses were performed using GraphPad Prism (version 8.0; GraphPad Software, La Jolla, CA, USA) software. Statistical significance was defined as a two-sided P-value of <0.05. Fisher’s exact tests were used to analyze categorical variables. The non-parametric Mann-Whitney test was used to calculate the difference in mTBI between groups. Time to recurrence and follow-up were calculated from the start of immunotherapy. Survival fractions were estimated using the Kaplan-Meier method and differences between groups were identified using the log-rank test. Multivariate Cox proportional hazards analysis was performed using SPSS software (version 22.0; STATA, College Station, TX, USA). The mutation heat map of tumor DNA was plotted using “Complex Heatmap” package in R software (version 3.4.1) (16).

## Results

### Demographic Information of NMIBC Patients

NMIBC patient characteristics are summarized in **Table 1**. This study enrolled 82 patients, including 13 (15.85%) female and 69 (84.15%) male patients. The median diagnostic age of patients was 63.5 years, with a range of 32 to 83 years. The patients with stage Ta and T1 represented 58.54% (48/82) and 41.46% (34/82) of the entire cohort, respectively. The proportions of low-grade (LG) and high-grade (HG) patients were 51.22% (42/82) and 48.78% (40/82), respectively. Twenty-five (30.49%) patients had a tumor size ≥3 cm.

### Mutational Profiling of Tumor DNA

**Figure 1:**
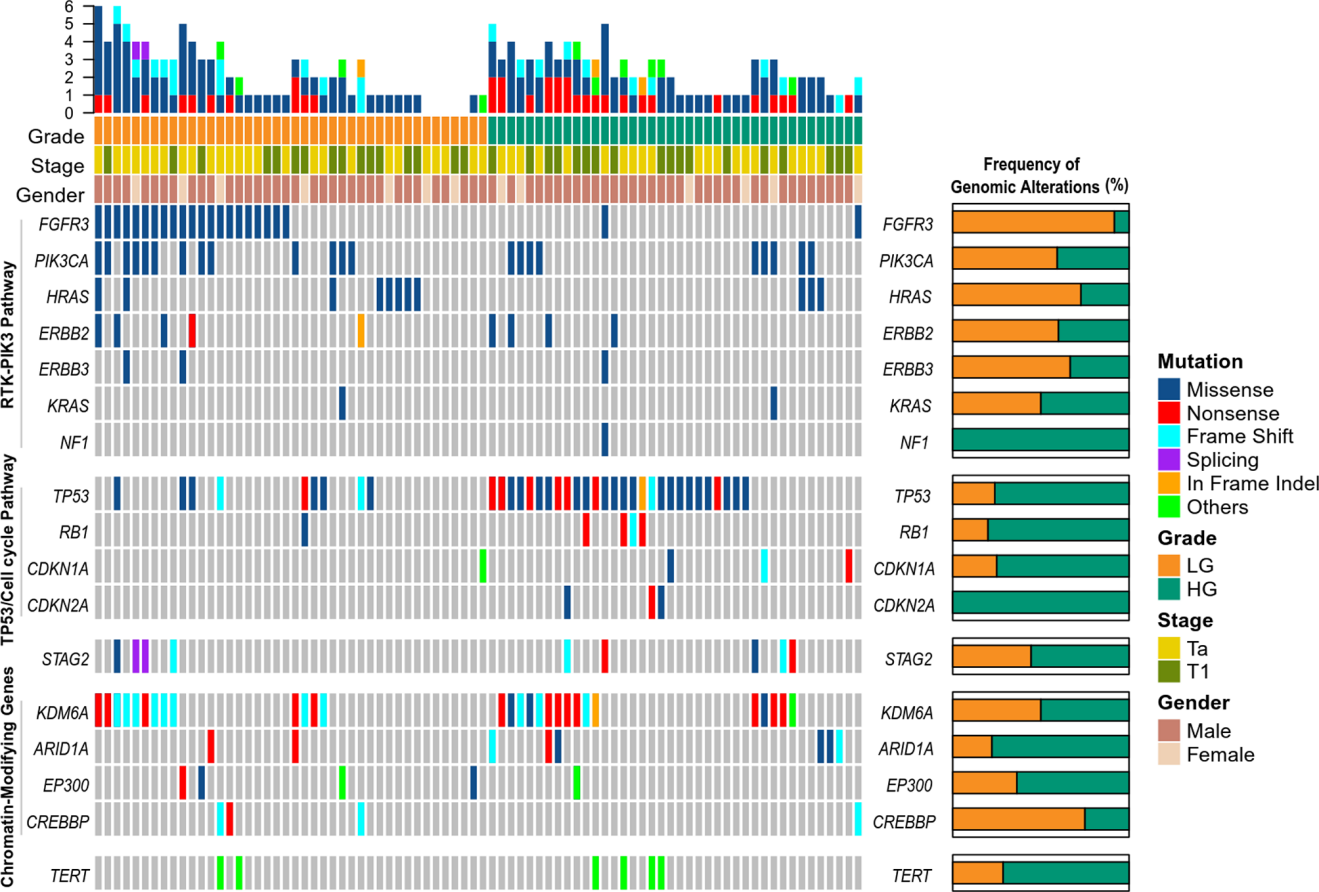
Genomic landscape of somatic mutations detected in tumor DNA from patients with non-muscle-invasive bladder cancer. LG, low grade; HG, high grade.

We detected 476 somatic mutations, including 416 Single nucleotide variants (SNVs) and 60 insertions and deletions (Indels), in tumor DNA from 82 patients (100%), with a median of 5 per sample (range, 1 to 18) (**Table S1**). The most frequently altered genes in tumors were TP53 (45.12%), KDM6A (35.37%), FGFR3 (28.04%), PIK3CA (26.83%), HRAS (13.41%), ERBB2 (10.98%), STAG2 (10.98%), ARID1A (9.76%) (**Figure 1**). To better define the prevalence of potentially actionable alterations, we conducted a pathway analysis of somatic variants. Alterations in the receptor tyrosine kinase/phosphatidylinositol 3-kinase pathway were identified more frequently in LG tumors in comparison to HG tumors (73.81 *vs.* 37.50%, p = 0.0017) (**Figure 1**). FGFR3 variants were seen more commonly in LG patients than in HG patients (50 *vs.* 5%, p < 0.0001) (**Figure 1**). In contrast, TP53 alterations were much higher in HG disease in comparison to LG disease (70 *vs.* 21.43%, p < 0.0001) (**Figure 1**). Variations in cell cycle regulation genes (RB1, CDKN1A, CDKN2A) were seen more commonly in HG-NMIBC than in LG-NMIBC (25.00 *vs.* 4.76%, p = 0.0122) (**Figure 1**).

**Figure 2:**
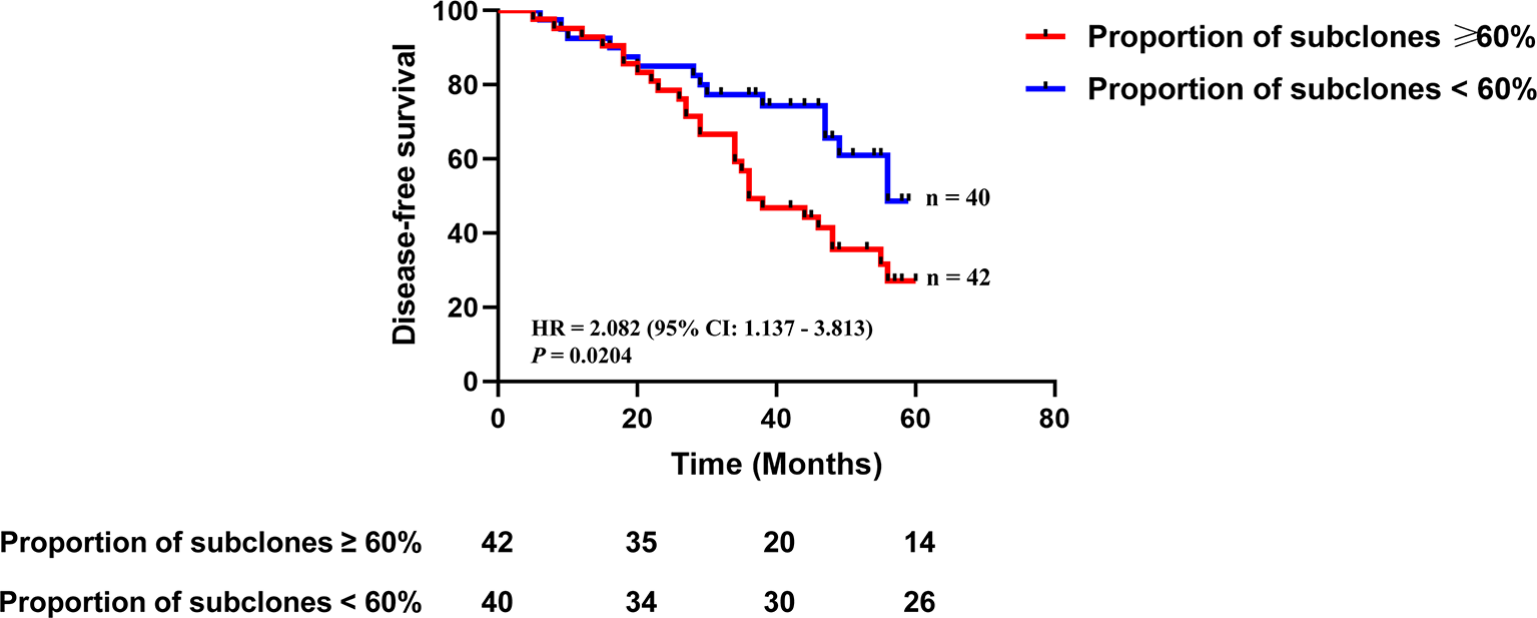
The association between intratumoral heterogeneity in tumor DNA and disease-free survival. Kaplan-Meier curves depict disease-free survival of 82 patients divided into high heterogeneity group (the proportion of subclones ≥60%) and low heterogeneity group (the proportion of subclones <60%). HR, hazard ratio. Differences between groups were identified using the log-rank test.

We analyzed clonality of mutations using PyClone software, which is based on a Bayesian clustering algorithm (17), using variants from 82 patients, which revealed 153 clonal mutations and 323 subclonal mutations. We defined intratumoral heterogeneity as the proportion of mutations that were inferred to be subclonal in a tumor sample. The proportion of subclones ranged from 0 to 94%, with a median of 60%. By using the median proportion of subclones as the cut-off value, patients were divided into high heterogeneity group (the proportion of subclones ≥60%, n = 42) and low heterogeneity group (the proportion of subclones <60%, n = 40). Patients with high heterogeneity had a significantly shorter disease-free survival (DFS) than those with low heterogeneity [median DFS 36 *vs.* 56 months, hazard ratio (HR) 2.082, 95% confidence interval (CI): 1.137–3.813, p = 0.0204] (**Figure 2**). Multivariable Cox analysis showed that heterogeneity was an independent prognostic indicator for DFS [HR 1.982 (95% CI: 1.024–3.835), p = 0.0420] (**Table 2**).

**Table 2:** Multivariate Cox proportional hazards analysis (n = 82).

Variable	Disease-free survival	
	HR (95% CI)	*P*-value
Age (≥60 *versus <*60)	1.296 (0.657–2.557)	0.455
Gender (male *versus* female)	0.832 (0.338–2.047)	0.689
Stage (Ta *versus* T1)	0.860 (0.443–1.668)	0.655
Size (≥3 cm *versus <*3* cm*)	1.761 (0.882–3.513)	0.109
Grade (LG *versus* HG)^‡^	0.938 (0.491–1.792)	0.845
Heterogeneity (≥60 *versus <*60%)	1.982 (1.024-3.835)	0.042^*^

^‡^LG, low grade; HG, high grade.

^*^P ≤ 0.05 considered statistically significant.

### The Relationship of Clinicopathological Features With ctDNA Detection and Detectability of Tumor-Derived Variations in Plasma

**Figure 3:**
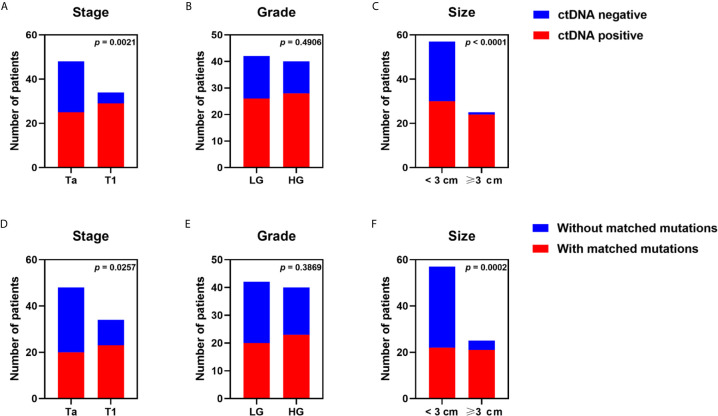
Effects of tumor stage, tumor grade, and tumor size on detectability of plasma ctDNA from patients with non-muscle-invasive bladder cancer. ctDNA detection rate of all samples grouped by **(A)** stage, **(B)** grade, **(C)** tumor size dichotomized at 3 cm. The detection rate of tumor variants in plasma ctDNA was compared in patients with **(D)** different tumor stages, **(E)** different tumor grades, **(F)** different tumor sizes. Each P-value was calculated with a Fisher’s exact test.

We detected 165 somatic mutations with a median of two per sample (range, 1 to 22) in plasma ctDNA from 54 patients (65.85%) (**Table S2**). Tumor DNA could be traced in matched ctDNA from 43 patients (52.44%). We evaluated the association between clinicopathological features and ctDNA detection. ctDNA was defined as positive if somatic mutations could be detected in plasma. The positive rate of ctDNA in T1 patients exceeded that in Ta patients (85.29 *vs.* 52.08%, p = 0.0021) (**Figure 3A**). ctDNA was more frequently detected in patients with tumor size ≥3 cm than in patients with tumor size <3 cm (96.00 *vs.* 52.63%, p < 0.0001) (**Figure 3C**).

The effects of clinicopathological features on detectability of tumor-derived variations in plasma were analyzed. Tumor-derived mutations were more often detected in plasma of patients with T1 stage in comparison to that of Ta patients (67.65 *vs.* 41.67%, p = 0.0257) (**Figure 3D**). Patients with tumor size ≥3 cm presented with more tumor-derived mutations in blood than patients with tumor size <3 cm (84.00 *vs.* 38.60%, p = 0.0002) (**Figure 3F**). However, we observed no significant association between tumor grade and ctDNA detection or detectability of tumor-derived variations in plasma (**Figures 3B, E**).

### Clinicopathological Associations of Detectability of Tumor-Derived Clonal or Subclonal Variations in Plasma

**Figure 4:**
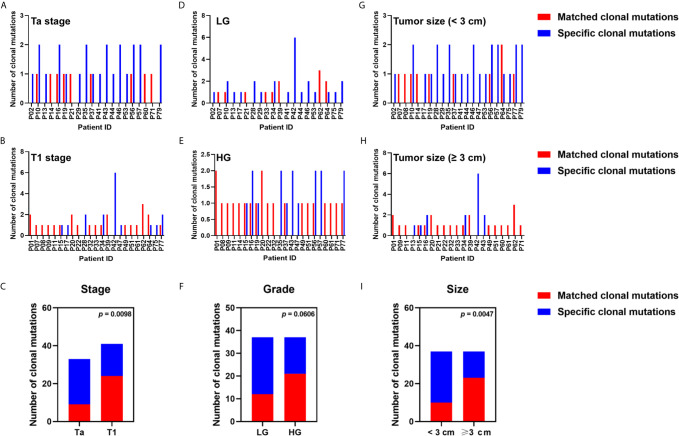
Relationship between clinicopathological features and the detection rate of tumor clonal variants in plasma ctDNA. The number of tumor clonal alterations detected or undetected in blood from patients with **(A)** Ta stage, **(B)** T1 stage, **(D)** low grade, **(E)** high grade, **(G)** tumor size ≥3 cm, **(H)** tumor size <3 cm, were displayed. The detection rate of tumor clonal mutation in plasma ctDNA was compared in patients with **(C)** different tumor stages, **(F)** different tumor grades, **(I)** different tumor sizes. Each P-value was calculated with a Fisher’s exact test.

To determine the relationship between clinicopathological features and detectability of tumor-derived clonal or subclonal variations in plasma, we analyzed clonality of mutations using variants from 43 patients with tumor-derived variations in plasma. We found 74 clonal and 202 subclonal mutations in tumor DNA from 43 patients (100%) and 39 patients (90.70%), respectively, while 77 clonal and 68 subclonal mutations were found in ctDNA from 43 patients (100%) and 20 patients (46.51%), respectively. Of 41 clonal mutations from tumor DNA in T1 patients, 24 (58.54%) was detected in matched ctDNA; however, the detection rate for patients with Ta stage was only 27.27% (9/33) (p = 0.0098) (**Figures 4A–C**). The detection rates for patients with tumor size ≥3 cm were significantly higher than that in patients with tumor size <3 cm (62.16 *vs.* 27.03%, p = 0.0047) (**Figures 4G–I**). The detection rates for HG patients exceeded that for LG patients (56.76 *vs.* 32.43%), although the difference did not reach statistical significance (p = 0.0606) (**Figures 4D–F**).

The detection of tumor-derived subclonal variations in blood was significantly associated with tumor stage (41.67% in T1 patients *vs.* 16.98% in Ta patients; p = 0.0002) (**Figures S1A–C**), tumor grade (39.39% in HG patients *vs.* 18.45% in LG patients; p = 0.0011) (**Figures S1D–F**), and tumor size (47.30% in patients with tumor size ≥3 cm *vs.* 17.97% in patients with tumor size <3 cm; p < 0.0001) (**Figures S1G–I**).

### Association Between Clinicopathological Features and mTBI

**Figure 5:**
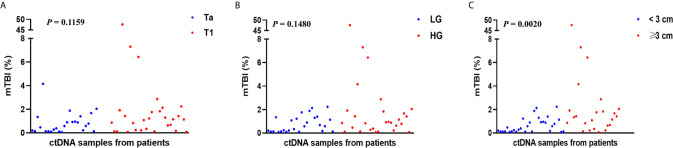
The association of clinicopathological features with mTBI. mTBI of all patients grouped by **(A)** tumor stage, **(B)** tumor grade, **(C)** tumor size dichotomized at 3 cm, was compared. Each P-value was calculated with a non-parametric Mann-Whitney test.

mTBI, a measure of molecular tumor burden, was calculated, and the relationship between clinicopathological features and mTBI was analyzed. We found a significant association between mTBI and tumor size. mTBI was significantly higher in patients with tumor size ≥3 cm than in patients with tumor size <3 cm (1.38 *vs.* 0.42%, p = 0.0020) (**Figure 5**).

### Correlation Between mTBI and Disease Recurrence

**Figure 6:**
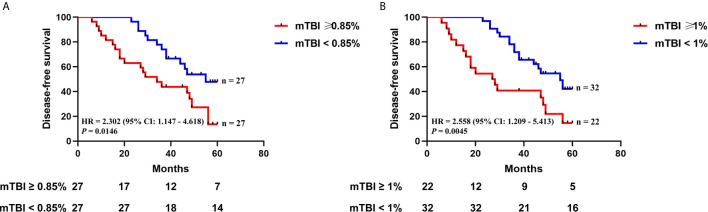
The relationship between mTBI in ctDNA and disease recurrence after TUR of bladder followed by immunotherapy. Kaplan-Meier curves depict disease-free survival of 54 patients receiving TUR of bladder followed by immunotherapy, stratified on the basis of mTBI threshold of **(A)** 0.85% (the median mTBI) or **(B)** 1%. HR, hazard ratio. Differences between groups were identified using the log-rank test.

Next, we evaluated whether mTBI correlated with DFS. ctDNA was detected in 54 patients who had received TUR of bladder followed by immunotherapy, and had been followed up for 6 to 60 months (median = 40.5 months). The median mTBI of ctDNA from these patients was 0.85%. The median DFS was shorter in patients with mTBI ≥ 0.85% in comparison to those with mTBI < 0.85% [median DFS 34 *vs.* 55 months, HR 2.302 (95% CI: 1.147–4.618), p = 0.0146] (**Figure 6A**). If using mTBI of 1% as the threshold, it was also significantly associated with worse outcome [median DFS of 27.5 months for mTBI ≥ 1% *vs.* 55 months for mTBI < 1%, HR 2.558 (95% CI: 1.209–5.413), p = 0.0045] (**Figure 6B**). Multivariable Cox analysis showed that mTBI was an independent prognostic indicator for DFS [HR 2.228 (95% CI: 1.095–4.531), p = 0.0270] (**Table 3**).

**Table 3:** Multivariate Cox proportional hazards analysis (n =54).

Variable	Disease-free survival	
	HR (95% CI)	*P*-value
Age (≥60 *versus <*60)	1.324 (0.595–2.948)	0.491
Gender (male *versus* female)	0.815 (0.285–2.335)	0.704
Stage (Ta *versus* T1)	0.704 (0.328–1.514)	0.370
Size (≥3 cm *versus <*3* cm*)	1.859 (0.853–4.053)	0.119
Grade (LG *versus* HG)	1.010 (0.464–2.201)	0.980
mTBI (≥0.85 *versus <*0.85%)^※^	2.228 (1.095–4.531)	0.027^*^

^※^mTBI, molecular tumor burden index.

^*^P ≤ 0.05 considered statistically significant.

## Discussion

Targeted sequencing has been applied for ctDNA detection in advanced bladder cancer patients (18–20). For instance, Christensen et al. reported that ctDNA assessment for early risk stratification, therapy monitoring, and early relapse detection in patients with localized advanced bladder cancer was feasible (18). However, targeted sequencing has not been performed in NMIBC patients, partly owing to the low detection of tumor DNA mutations in blood in NMIBC patients. This study is the first to perform targeted sequencing of ctDNA for NMIBC patients. Our cohort included NMIBC patients at stage Ta and T1. Consistent with previous reports (12), we found that ctDNA from plasma samples could be detected in NMIBC patients with Ta stage, with a detection rate of 52.08%. These results showed that DNA may be released into circulation by some transporter, such as exosomes, even though Ta tumors had an intact basal membrane. The detection rate of ctDNA and the concordance rate of somatic mutations between ctDNA and tumor DNA were higher in T1 patients than in Ta patients, suggesting that invasive state of NMIBC could increase release of ctDNA into blood, and ctDNA levels are reflecting invasiveness of disease. We also found that the larger tumor size was significantly associated with the higher ctDNA detection rate and the higher concordance rate of somatic mutations between ctDNA and tumor DNA. These results showed that larger tumor size represented higher tumor burden, leading to increase in ctDNA release into circulation.

Ma et al. reported that mTBI had advantage in assessing therapeutic response compared with single gene mutations (21). Wang et al. found that mTBI could serve as a predictive biomarker of treatment outcome for advanced gastric cancer patients receiving chemotherapy (22). A higher mTBI in baseline ctDNA was a risk factor for prognosis. Thus, we evaluated whether mTBI could predict treatment outcome in NMIBC patients receiving TUR of bladder followed by immunotherapy. We found that higher mTBI was associated with shorter DFS in such patients, and could be an independent prognostic indicator for DFS.

Consistent with previous studies (23, 24), the pathway analysis of somatic variants for tumor DNA showed that alterations in either FGFR3 or the receptor tyrosine kinase/phosphatidylinositol 3-kinase pathway were identified more frequently in LG-NIMBC in comparison to HG-NIMBC, whereas variations in either TP53 or cell cycle regulation genes were more common in HG disease than in LG disease. These results suggest that the development of LG-NMIBC and HG-NMIBC may be controlled by the receptor tyrosine kinase/phosphatidylinositol 3-kinase pathway and TP53/cell cycle regulation genes, respectively. Thus, LG-NMIBC and HG-NMIBC patients should be treated with different targeted therapies. For instance, FGFR3 inhibitor may not be effective for HG-NMIBC.

The study by Christensen et al. utilized ddPCR analyses and screened ctDNA for FGFR3 and PIK3CA hotspot mutations in NMIBC (13). This study had an obvious limitation: variants in FGFR3 gene tend to be detected in LG-NMIBC. In our study, FGFR3 and PIK3CA alterations were seen in 28.04 and 26.83% of NMIBC patients, respectively. Thus, the analyses targeting FGFR3 and PIK3CA hotspot mutations can only cover a small proportion of patients with NMIBC.

Liu et al. defined intratumoral heterogeneity as the proportion of mutations that were subclonal in a tumor sample, and found that high post-treatment heterogeneity was associated with decreased overall survival for MIBC patients undergoing neoadjuvant cisplatin-based chemotherapy (25). We found that high pre-treatment heterogeneity was associated with worse DFS for NMIBC patients receiving TUR of bladder followed by immunotherapy.

In summary, we found that the detection rate of ctDNA and the concordance of somatic variants between tumor DNA and plasma ctDNA were both significantly associated with tumor stage and tumor size. Furthermore, mTBI in ctDNA could serve as an independent prognostic indicator of DFS for NMIBC patients receiving TUR of bladder followed by immunotherapy, and intratumoral heterogeneity in tumor DNA could also independently predict treatment outcome for such patients.

## Data Availability Statement

The original contributions presented in the study are included in the article/**Supplementary Material**. Further inquiries can be directed to the corresponding author.

## Ethics Statement

The studies involving human participants were reviewed and approved by Shenzhen Second People’s Hospital. The patients/participants provided their written informed consent to participate in this study.

## Author Contributions

Study concepts: JZ, DD, and AT. Study design: DD, JT, JZ, and AT. Data acquisition: LL, JB, and YX. Quality control of data and algorithms: LL, JB, and YX. Data analysis and interpretation: DD, JT, JZ, and ZW. Statistical analysis: DD and LL. Manuscript preparation: JZ and DD. Manuscript editing: JZ and DD. Manuscript review: DD, JT, JZ, LL, JB, ZW, YX, and AT. All authors contributed to the article and approved the submitted version.

## Funding

This work was supported by the National Science Foundation of China (Grant numbers 81772736, 32000244), Shenzhen Science and Technology Innovation Commission (Grant numbers 20190726095103499, JSGG20191231141403880, and JSGG20200102165803939), Lanzhou University Second Hospital (Grant number CY2017-BJ16), Industry Planning Project of Health Department of Gansu Province (Grant number GWGL2013-30), Lanzhou Talent Innovation and Entrepreneurship Project (Grant number 2019-RC-37), and Guangdong Natural Science Foundation (Grant number 2018A030313872).

## Conflict of Interest

Authors LL, JB and YX were employed by company Geneplus-Beijing Institute, Beijing, China.

The remaining authors declare that the research was conducted in the absence of any commercial or financial relationships that could be construed as a potential conflict of interest.

## Supplementary Material

Supplementary Figure 1 The effects of clinicopathological features on the detection rate of tumor subclonal variations in plasma ctDNA. The number of tumor subclonal mutations detected or undetected in blood from patients with **(A)** Ta stage, **(B)** T1 stage, **(D)** low grade, **(E)** high grade, **(G)** tumor size ≥ 3 cm, **(H)** tumor size < 3 cm, were displayed. The detection rate of tumor subclonal mutation in plasma ctDNA was compared in patients with **(C)** different tumor stages, **(F)** different tumor grades, **(I)** different tumor sizes. Each P-value was calculated with a Fisher’s exact test.
