# Supplementary figures and images for: Circulating Tumor DNA Analyses Predict Disease Recurrence in Non-Muscle-Invasive Bladder Cancer
Source: Front Oncol. 2021 Apr 28;11:657483. doi: 10.3389/fonc.2021.657483 (PMC8114939; doi:10.3389/fonc.2021.657483)

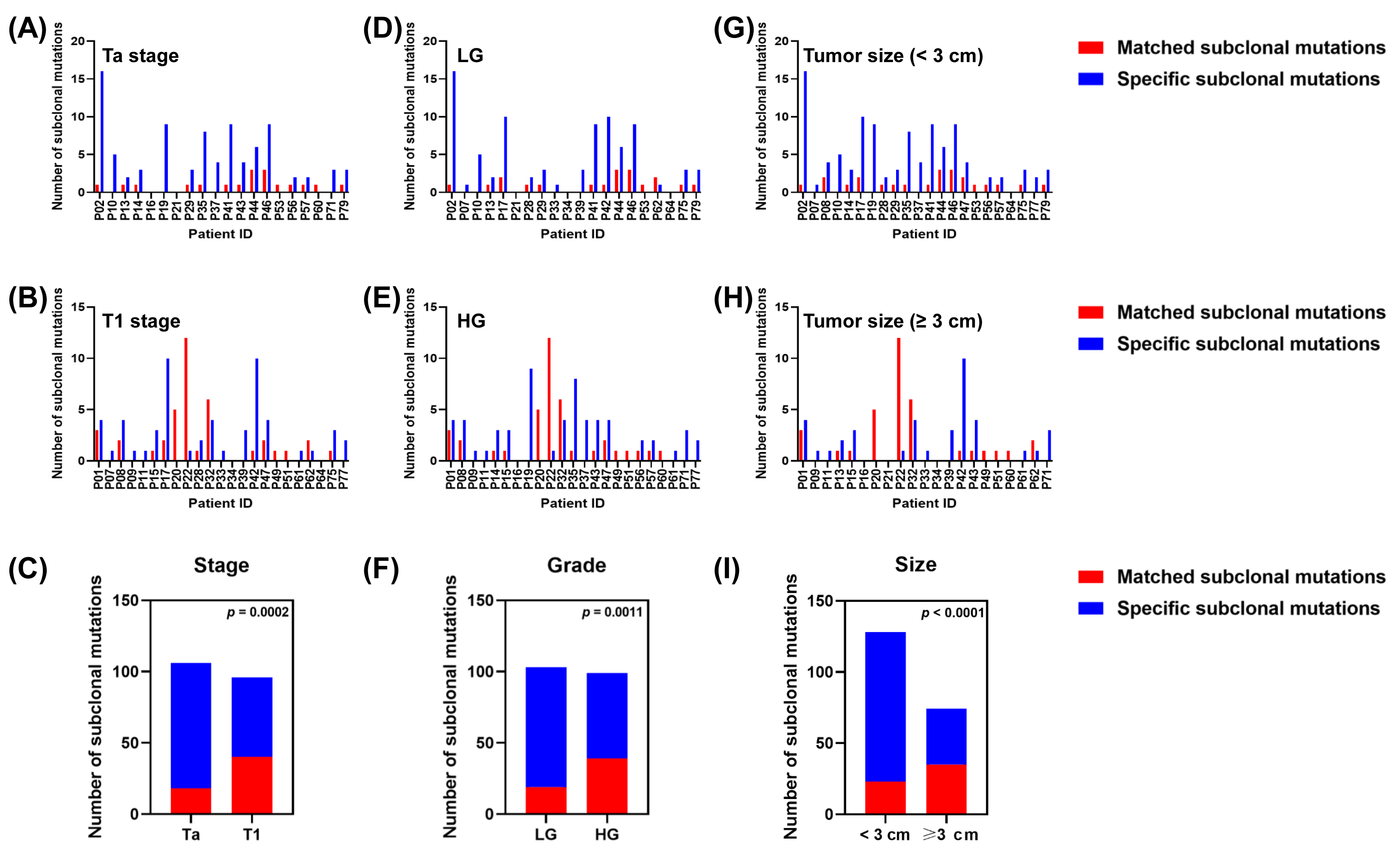

Supplement: Supplementary Figure 1 — The effects of clinicopathological features on the detection rate of tumor subclonal variations in plasma ctDNA. The number of tumor subclonal mutations detected or undetected in blood from patients with (A) Ta stage, (B) T1 stage, (D) low grade, (E) high grade, (G) tumor size ≥ 3 cm, (H) tumor size < 3 cm, were displayed. The detection rate of tumor subclonal mutation in plasma ctDNA was compared in patients with (C) different tumor stages, (F) different tumor grades, (I) different tumor sizes. Each P-value was calculated with a Fisher’s exact test. [file Image_1.tif]
